# Supplementary material for: The structural and functional investigation into an unusual nitrile synthase
Source: Nat Commun. 2023 Nov 16;14:7425. doi: 10.1038/s41467-023-43285-0 (PMC10654658; doi:10.1038/s41467-023-43285-0)
Supplement: Supplementary file 1 — Supplementary Information [file 41467_2023_43285_MOESM1_ESM.docx]

**Supplementary materials**

**The structural and functional investigation into an unusual nitrile synthase**

Hao Li^1,4^, Jian-Wen Huang^1,4^, Longhai Dai^1,4^, Haibin Zheng^1^, Si Dai^1^, Qishan Zhang^1^, Licheng Yao^2^, Yunyun Yang^1^, Yu Yang^1^, Jian Min^1^, and Rey-Ting Guo^1,3^*, Chun-Chi Chen^1,3^*

^1^State Key Laboratory of Biocatalysis and Enzyme Engineering, Hubei Hongshan Laboratory, Hubei Collaborative Innovation Center for Green Transformation of Bio-Resources, Hubei Key Laboratory of Industrial Biotechnology, School of Life Sciences, Hubei University, Wuhan 430062, PR China

^2^Hubei Gongtong Steroid Drug Research Institute, Wuhan 430073, PR China

^3^Department of Immunology and Pathogen Biology, School of Basic Medical Sciences, Hangzhou Normal University, Hangzhou 311121, PR China

^4^These authors contributed equally

*Corresponding authors. E-mail addresses: [guoreyting@hubu.edu.cn](mailto:guoreyting@hubu.edu.cn) (R.-T. Guo), [ccckate0722@hubu.edu.cn](mailto:ccckate0722@hubu.edu.cn) (C.-C. Chen).

**Figure**

**
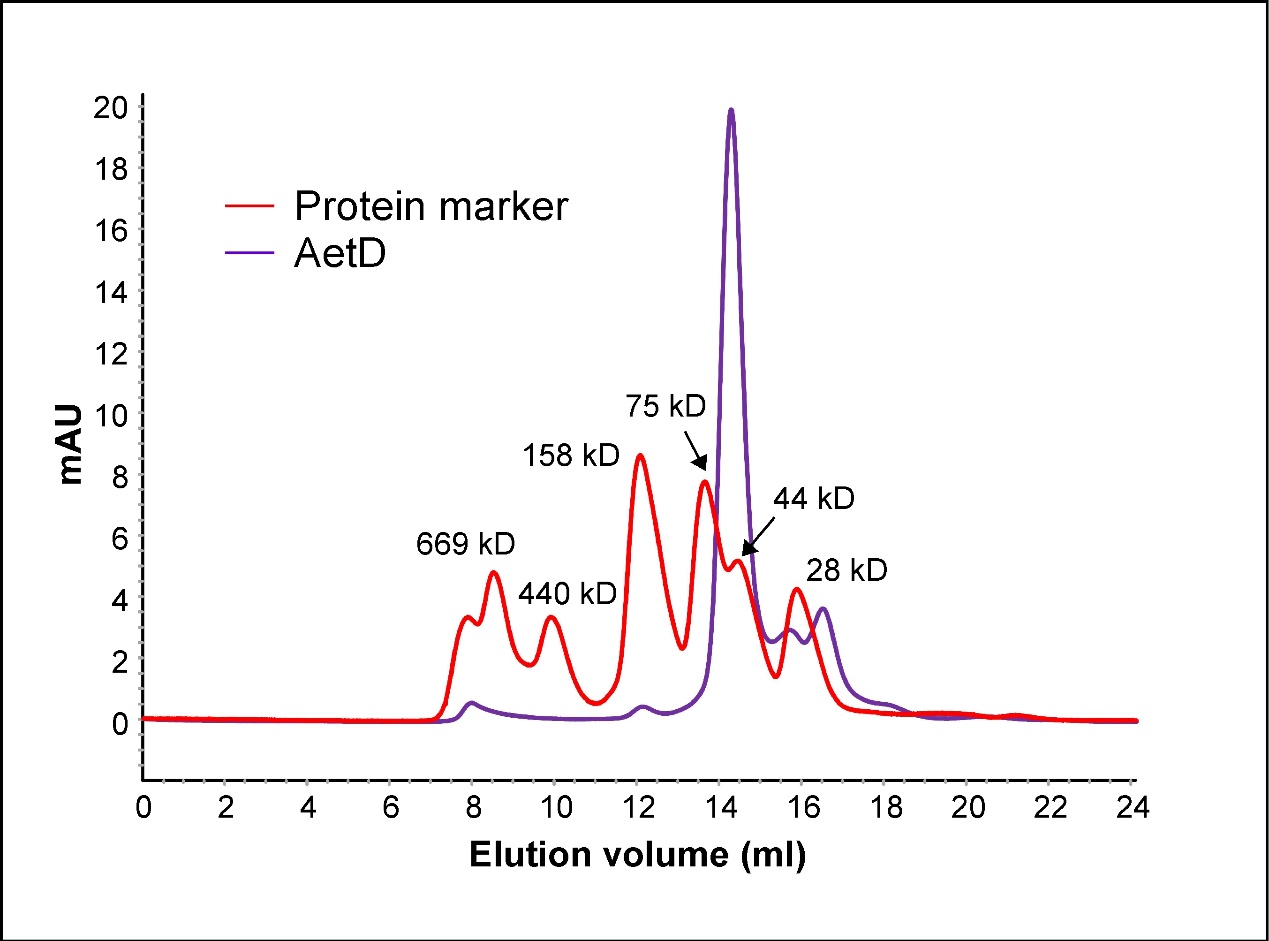
**

**Supplementary Fig. 1. Size exclusion chromatography analysis of AetD**. Red and purple chromatogram traces represent the standard protein markers and recombinant protein of AetD, respectively. The theoretical molecular weight for monomeric and homodimeric AetD is 27.97 kDa and 55.94 kDa, respectively. The calculated molecular weight of the major peak of AetD was ~ 55.8 kDa.

**
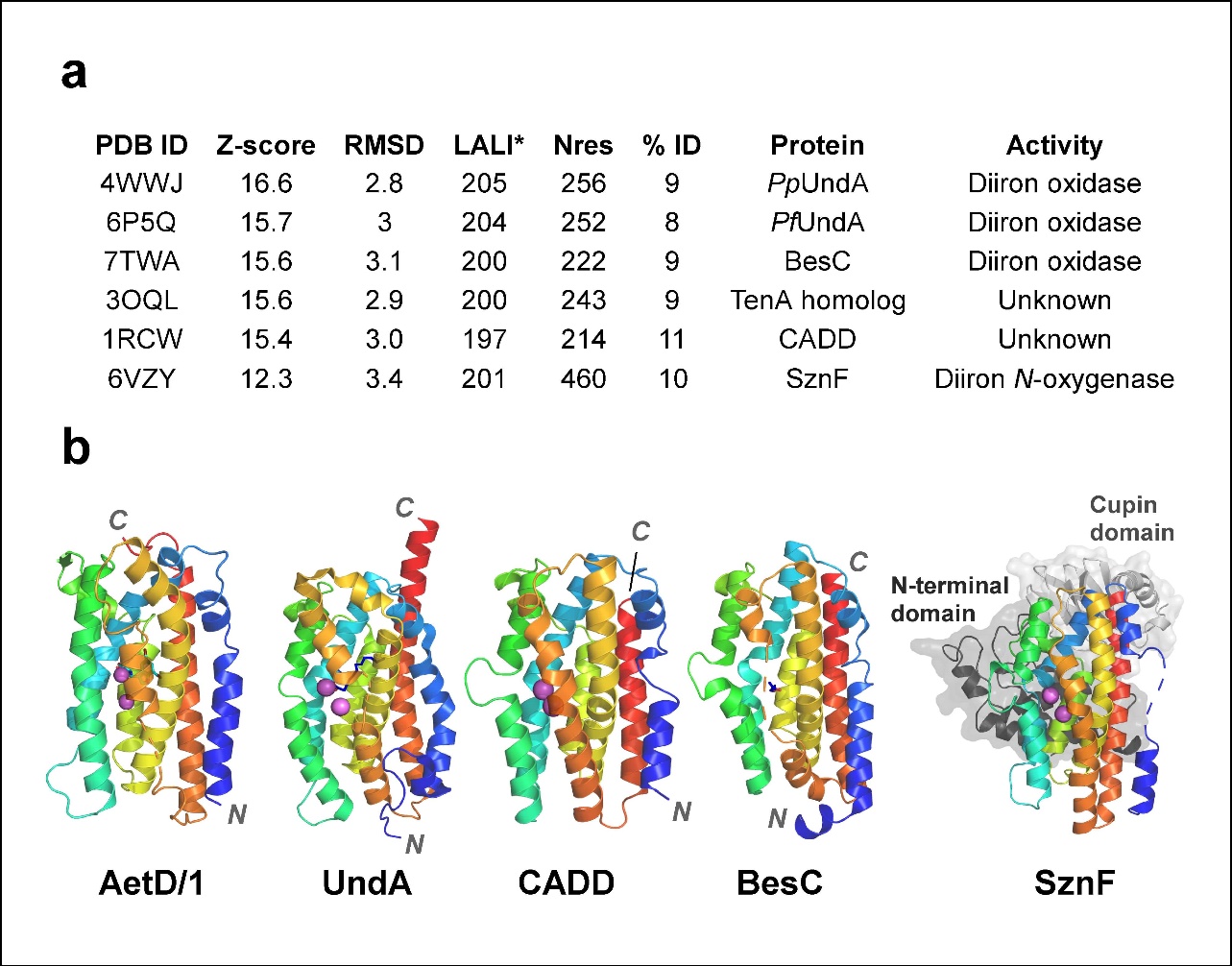
**

**Supplementary Fig. 2. Structures of AetD and several HO-fold proteins**. **a.** Partial results of AetD-homologous structure searching with the DALI server. *Pp*UndA and *Pf*UndA, UndA from *Pseudomonas protegens* and *P. fluorescenes*, respectively. *LALI, number of equivalent residues. **b.** The overall structures of AetD complex with **1**, UdnA from *P. fluorescenes* (PDB ID, 6P5Q) [http://doi.org/10.2210/pdb6P5Q/pdb], CADD from *Chlaymydia trachomatis* (PDB ID, 1RCW) [https://doi.org/10.2210/pdb1RCW/pdb], BesC from *Streptomyces cattleya* (PDB ID, 7TWA) [https://doi.org/10.2210/pdb7TWA/pdb] and the HO-like domain of SznF from *Streptomyces achromogenes* (PDB ID, 6VZY) [https://doi.org/10.2210/pdb6VZY/pdb] are shown in cartoon models colored in rainbow. The N-terminal domain and cupin domain of SznF are displayed in cartoon/surface model in dark and light gray, respectively. Fe ions and bound ligands are shown in pink spheres and blue sticks.


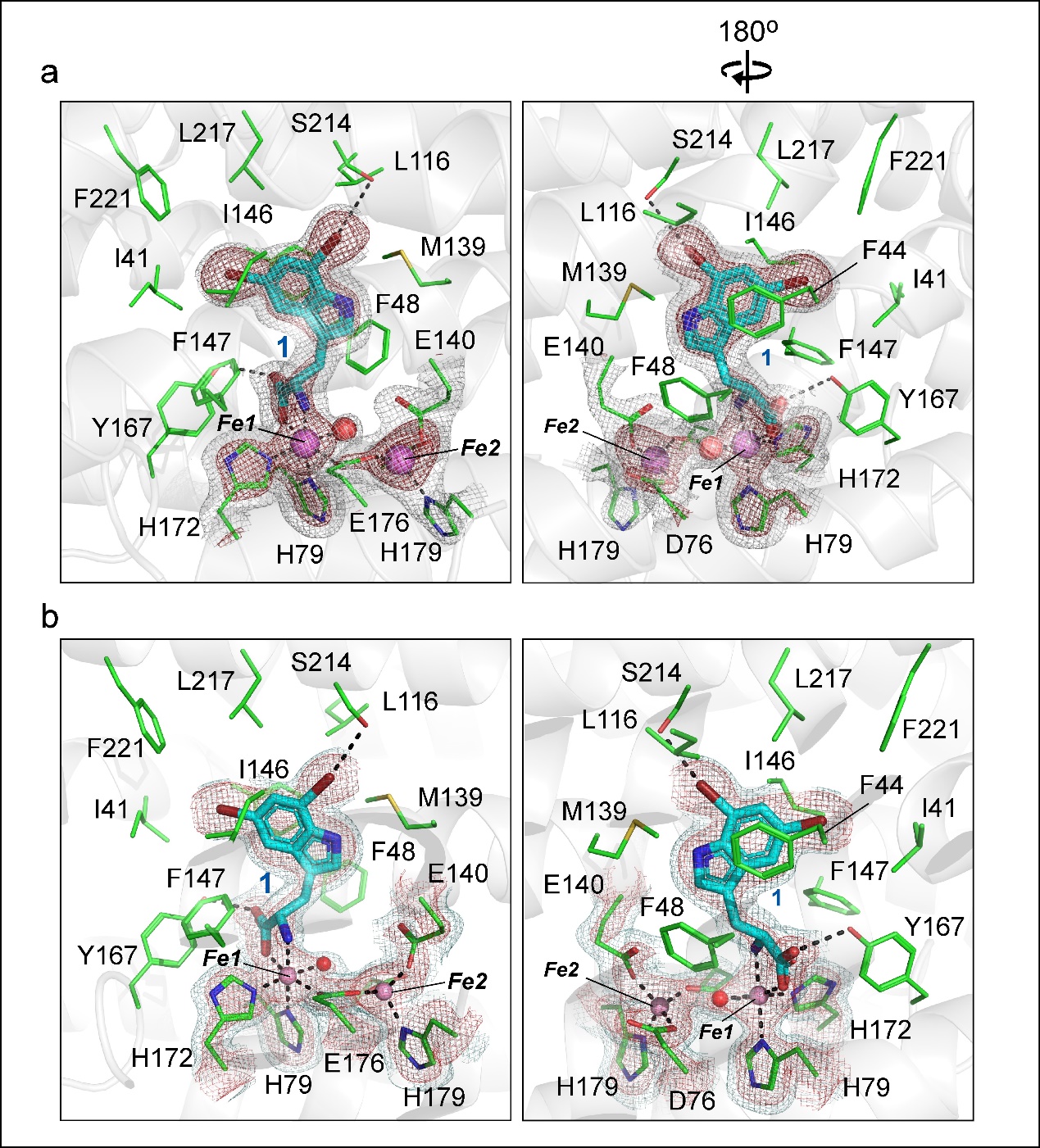


**Supplementary Fig. 3. Electron maps of ligands bound in the AetD/1/Fe complex structure**. The (a) 2*F*_o_-*F*_c_ electron density maps and (b) *F*_o_-*F*_c_ omit maps of **1** (cyan stick), Fe ions (pink spheres), the Fe-coordinating water molecule (red sphere) and Fe-coordinating residues (lines) are contoured at 3.0 σ (red mesh) and 1.0 σ (lightcyan mesh), respectively. The protein models and residues are shown in as described in **Fig. 2**.


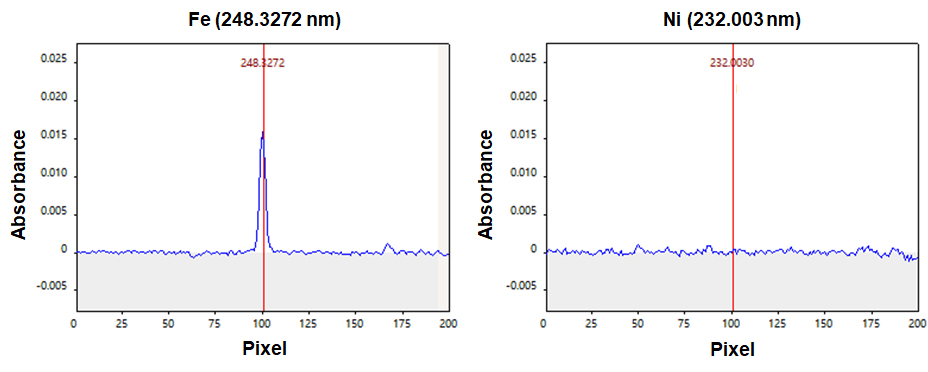
**Supplementary Fig. 4. Atomic absorption spectrometric analyses.** The purified protein of AetD was subjected to atomic absorption spectroscopic analyses by monitoring 248.3272 nm and 232.003 nm wavelength to detect Fe and Ni ion, respectively.


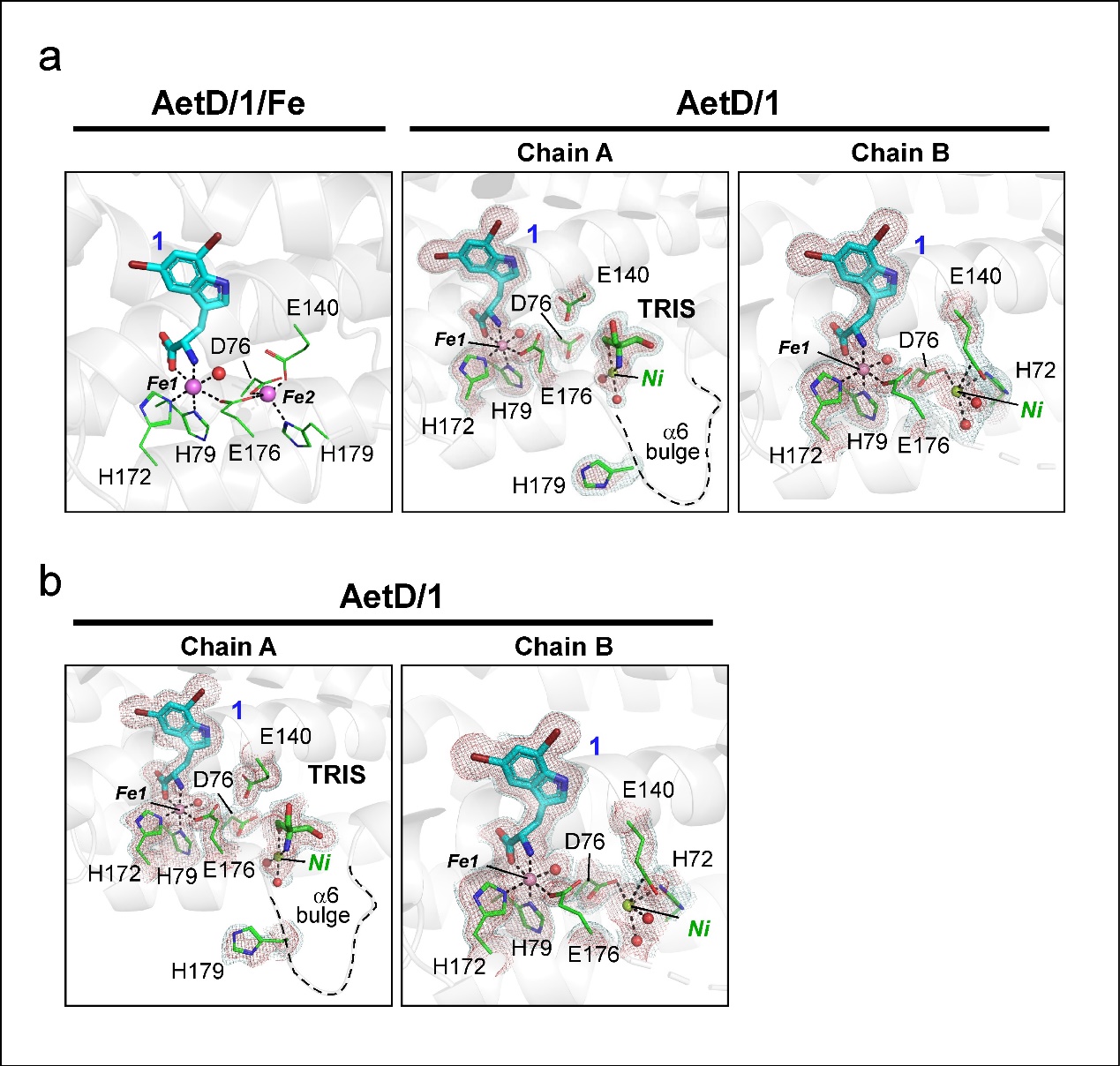


**Supplementary Fig. 5. Metal ion coordination status in AetD/1 complex structures.** The enzyme-ligand interaction and metal ion coordination networks in AetD complex structures are displayed. (a) Chain A in AetD/**1**/Fe crystals and both chains (chain A and chain B) in AetD/**1** crystals are shown. The bulge regions in helix α6 in chain A of AetD/**1** crystal is traced by dashed curves, whose counterparts in chain B was not observed owing to the lack of electron density. The metal coordination status in chain B of AetD/**1**/Fe is identical to that in chain A, thus only one chain is displayed here. The 2*F*_o_-*F*_c_ electron density maps of protein residues (green lines), bound ligands (sticks), waters (red spheres) and metal ions (violet for Fe and lime for Ni) in AetD/**1** are contoured at 2.0 σ (red mesh) and 1.0 σ (lightteal mesh). Dashed lines linking the metal ions and protein residues are coordinate bonds. (b) The *F*_o_-*F*_c_ omit maps of protein residues, bound ligands, waters and metal ions in AetD/**1** are contoured at 2.0 σ (red mesh) and 1.0 σ (lightteal mesh). The model presentations are as described in panel (a).


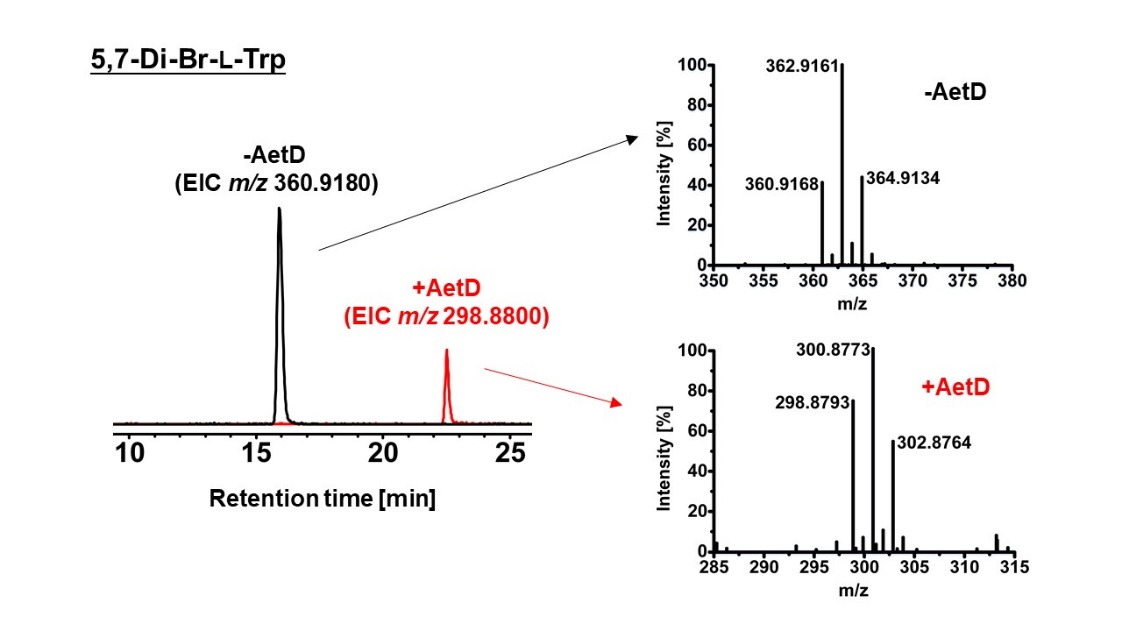

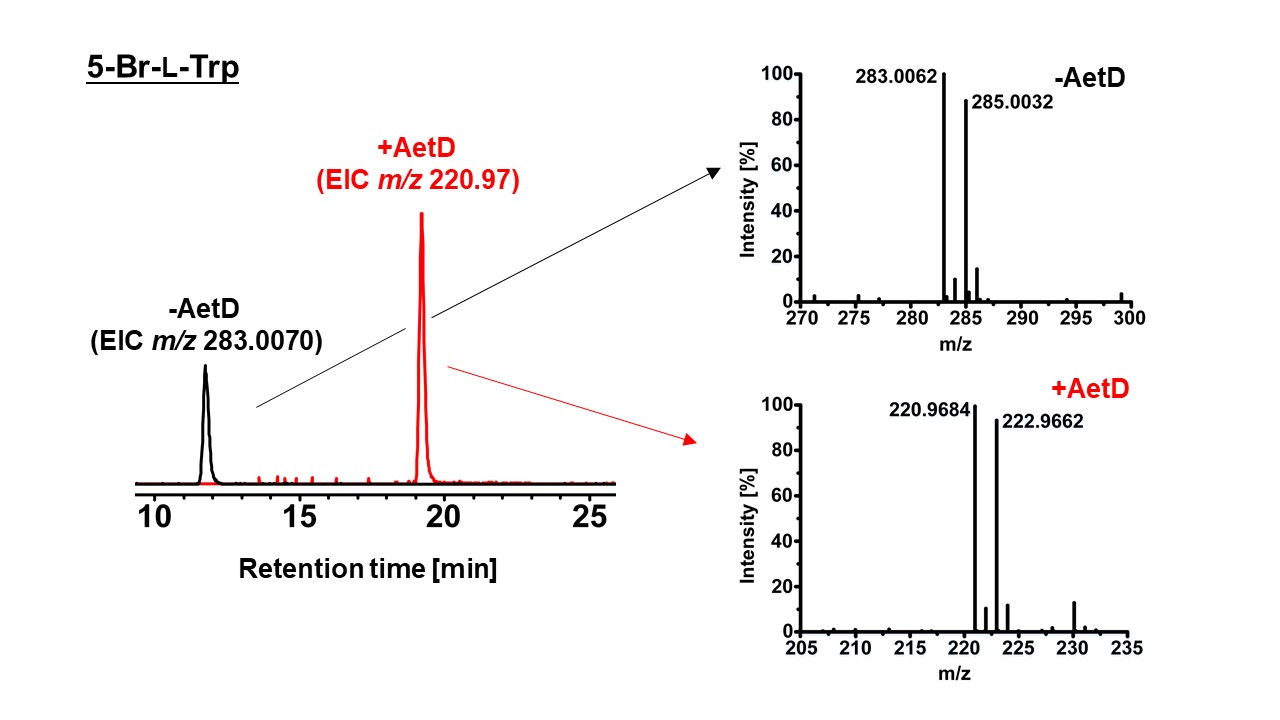

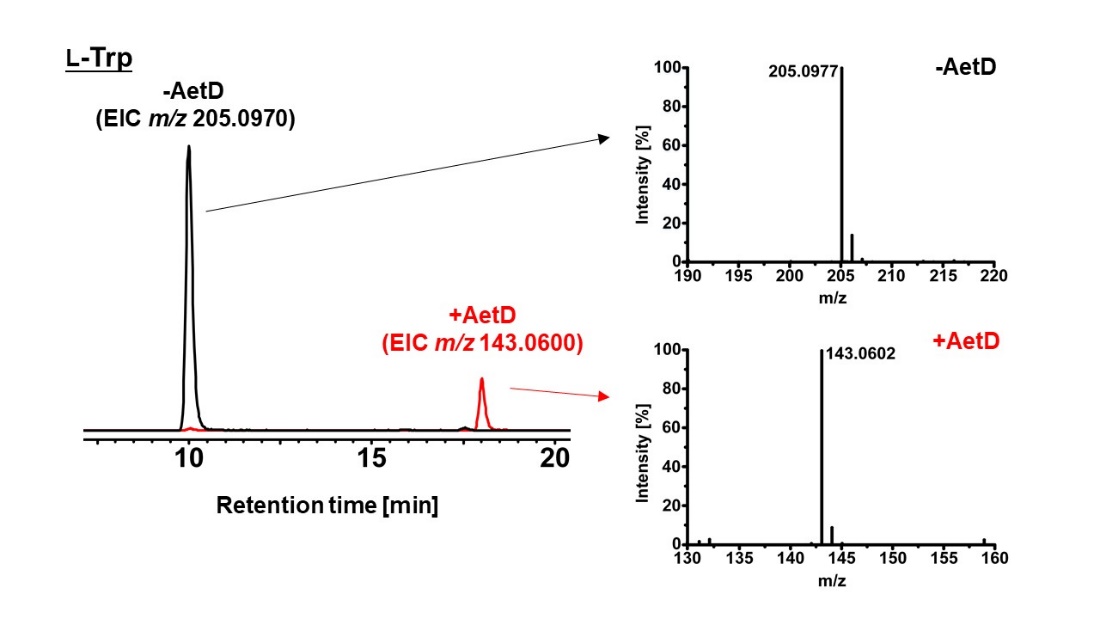


**Supplementary Fig. 6. LC-MS analysis of AetD-catalyzed reaction.** Extracted ion chromatograms (EICs) of (upper) AetD-transformed products of 5,7-di-Br-L-Trp (**1**), (middle) 5-Br-L-Trp (**2**) and (lower) L-Trp (**3**) in positive ionization mode. Black traces indicate the reactions without containing AetD.


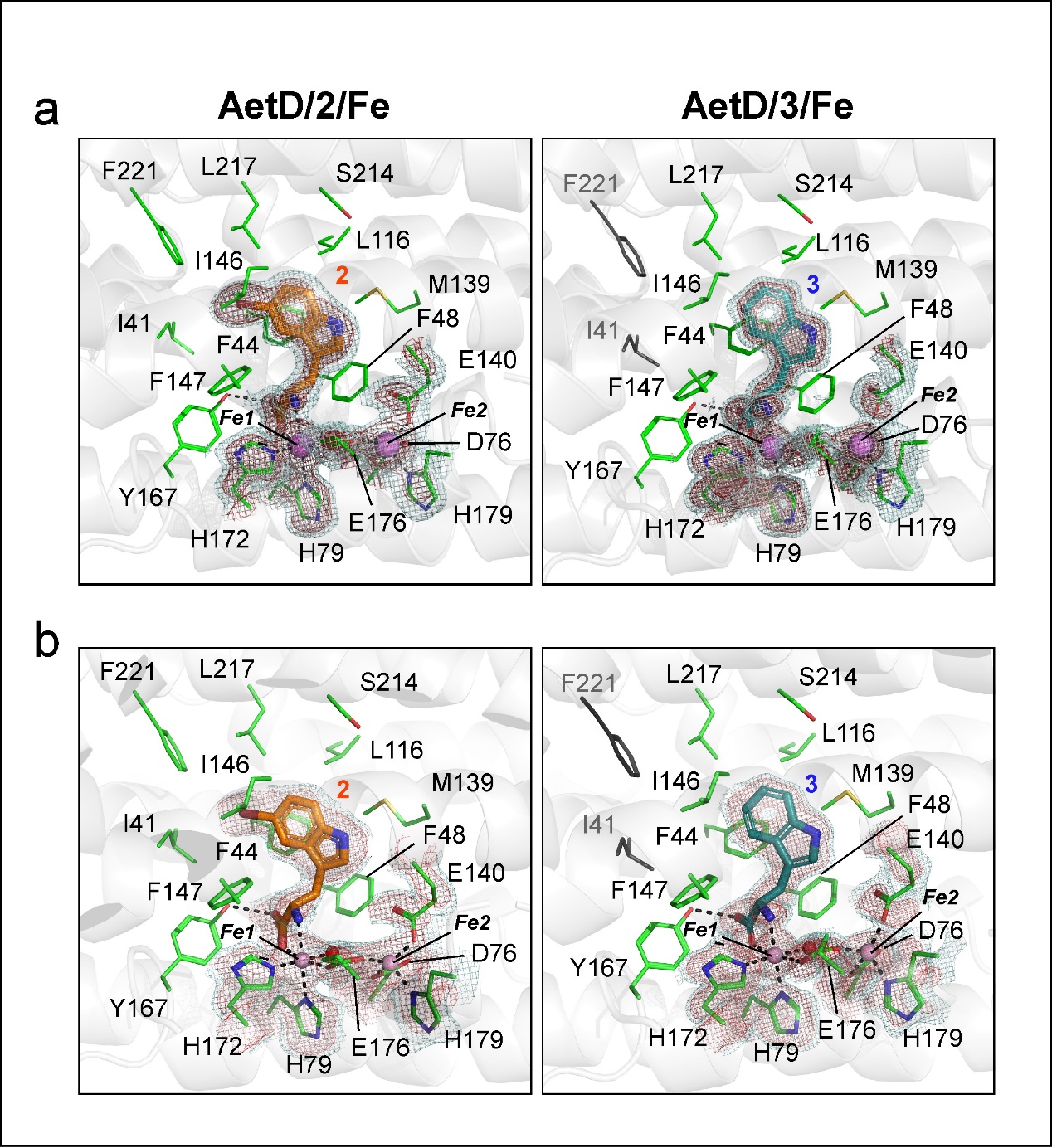


**Supplementary Fig. 7. Electron maps of ligands bound in the structures of AetD in complex with 2 and 3**. The (a) 2*F*_o_-*F*_c_ electron density maps and (b) *F*_o_-*F*_c_ omit maps of the bound ligands (stick), Fe ions (pink spheres), the coordinating water molecules (red spheres) and Fe-coordinating residues (line) are contoured at 2.5 σ (red mesh) and 1.0 σ (lightcyan mesh), respectively. The protein models and residues are shown in as described in **Supplementary Fig. 3**.


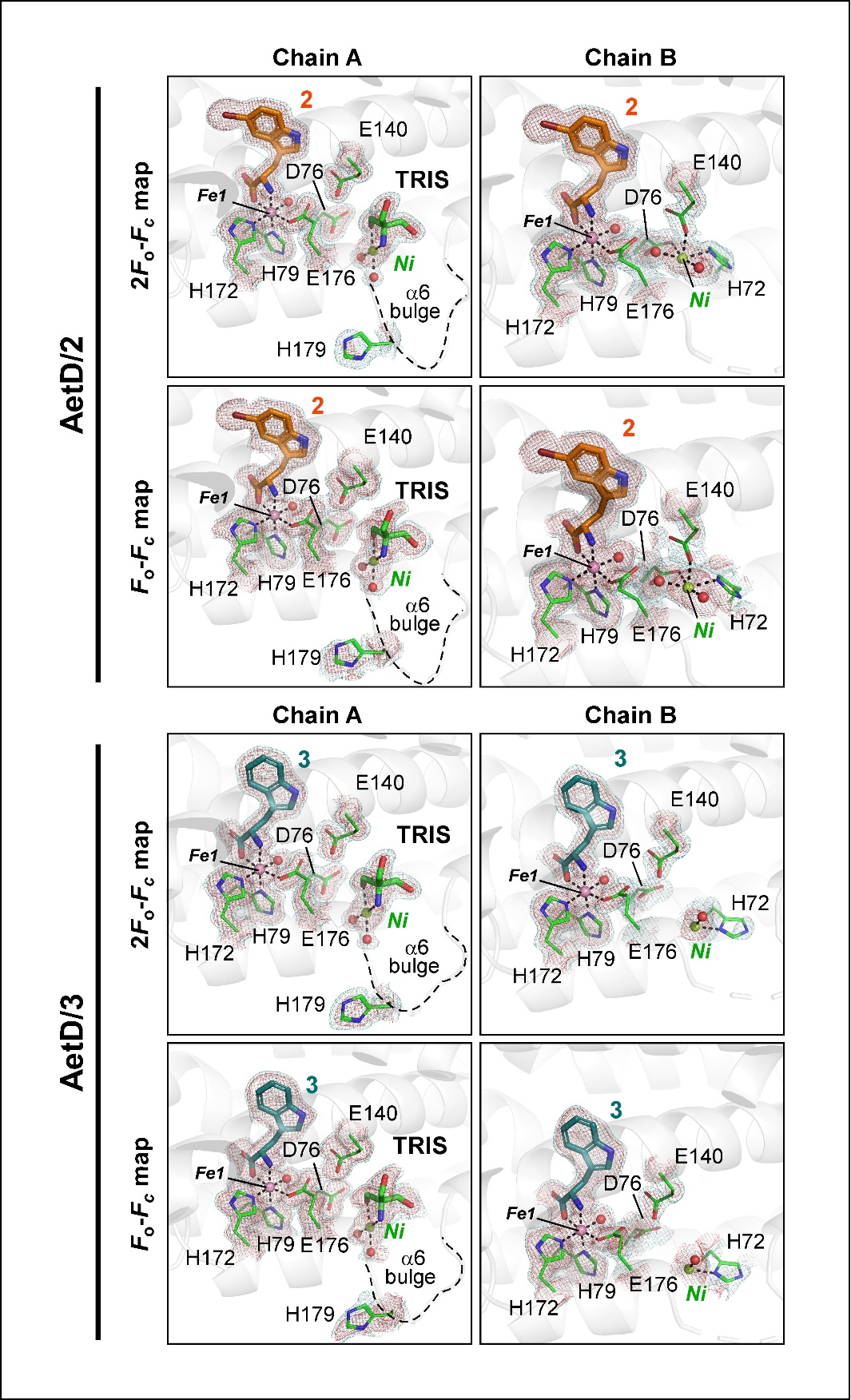


**Supplementary Fig. 8. Metal ion coordination status in AetD/2 and AetD/3 complex structures.** The enzyme-ligand interaction and metal ion coordination networks in AetD complex structures are displayed. Chain A and chain B in AetD/**2** and AetD/**3** are shown. The bulge regions and disordered region in helix α6 are displayed as in **Supplementary Fig. 5**. The 2*F*_o_-*F*_c_ electron density maps and *F*_o_-*F*_c_ omit maps of protein residues (green lines), bound ligands (sticks), waters (red spheres) and metal ions (violet for Fe and lime for Ni) in two complexes are contoured at 2.0 σ (red mesh) and 1.0 σ (lightteal mesh), respectively. Dashed lines linking the metal ions and protein residues are coordinate bonds.


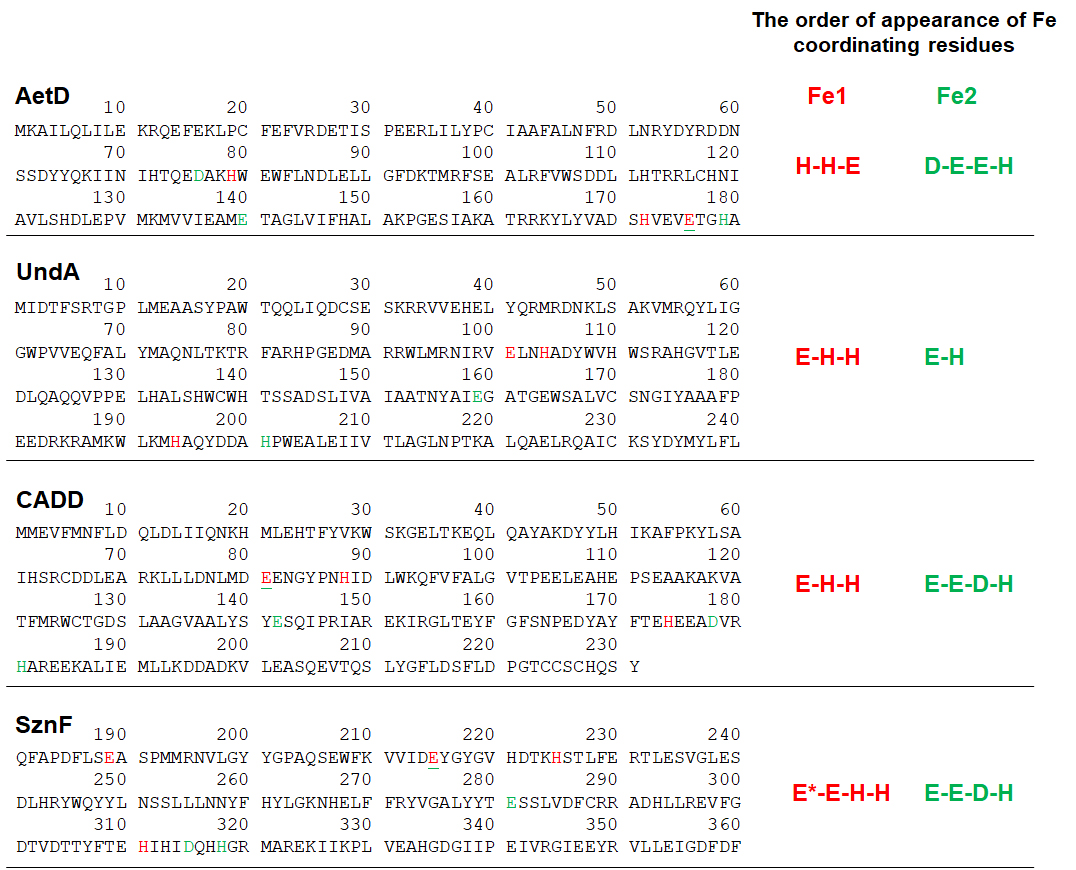


**Supplementary Fig. 9. Partial protein sequences of AetD and several HO-like fold proteins**. The partial protein sequences of AetD, UndA from *Pseudomonas* (PDB ID, 6P5Q) [http://doi.org/10.2210/pdb6P5Q/pdb], CADD from *C. trachomatis* (GenBank accession no. WP_009871978) [https://www.ncbi.nlm.nih.gov/protein/WP_009871978] and the HO-like domain of SznF from *S. achromogenes* (PDB ID, 6VZY) [https://doi.org/10.2210/pdb6VZY/pdb] that contain Fe ion-coordinating residues are displayed. Residues serve to coordinate the Fe1 and Fe2 are colored in red and green, respectively. For E176 in AetD, E81 in CADD and E215 in SznF that participate in both Fe1 and Fe2 coordination, red-colored characters with green underlines are used for the labeling. E*, the SznF-unique E189, which is absent in other HO-like proteins.


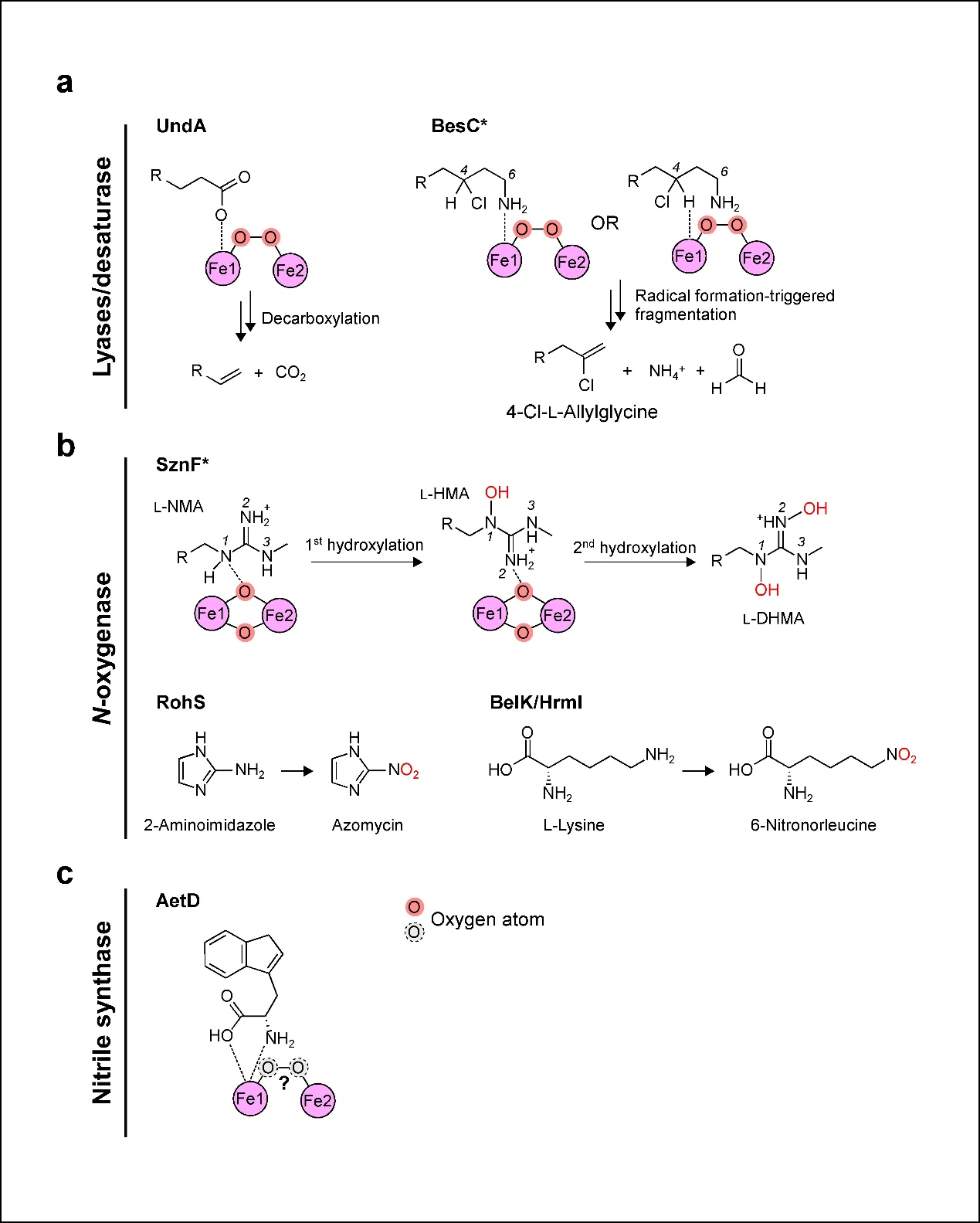


**Supplementary Fig. 10. Catalytic reaction of HO-like diiron enzymes**. The mechanism of UndA was proposed based on crystallographic analyses^[1](#_ENREF_1)^. Note that the substrate-binding pose and reaction process of BesC and SznF were proposed based on spectroscopic analysis^[2](#_ENREF_2)^ and molecular dynamics and QM/MM calculations^[3](#_ENREF_3)^, respectively (marked by asterisks). For SznF, the substrate-binding modes in the sequential hydroxylations displayed herein are also postulated models. L-NMA, N^ω^-methyl-L-arginine; L-HMA, N^δ^-hydroxy-N^ω^-methyl-L-Arg; L-DHMA, N^δ^,N^ω^-dihydroxy-N^ω^-methyl-L-Arg.**Table**

**Supplementary Table 1. Amino acid sequences of recombinant AetD and AetF used in this study**

| AetD | MSDKIIHLTDDSFDTDVLKADGAILVDFWAEWCGPCKMIAPILDEIADEYQGKLTVAKLNIDQNPGTAPKYGIRGIPTLLLFKNGEVAATKVGALSKGQLKEFLDANLAGSGSGHMHHHHHHSSGLVPRGSGMKETAAAKFERQHMDSPDLGTDDDDKAMEHHHHHHENLYFQGAGAGAGAGAGMKAILQLILEKRQEFEKLPCFEFVRDETISPEERLILYPCIAAFALNFRDLNRYDYRDDNSSDYYQKIINIHTQEDAKHWEWFLNDLELLGFDKTMRFSEALRFVWSDDLLHTRRLCHNIAVLSHDLEPVMKMVVIEAMETAGLVIFHALAKPGESIAKATRRKYLYVADSHVEVETGHAVGTENIITILEQTQLSSEQEEKAKEIVNKVFQWSTNLIGEFERYVKAHRSEKAQPTAAY |
| --- | --- |
| AetF | MSDKIIHLTDDSFDTDVLKADGAILVDFWAEWCGPCKMIAPILDEIADEYQGKLTVAKLNIDQNPGTAPKYGIRGIPTLLLFKNGEVAATKVGALSKGQLKEFLDANLAGSGSGHMHHHHHHSSGLVPRGSGMKETAAAKFERQHMDSPDLGTDDDDKAMEHHHHHHENLYFQGAGAGAGAGAGMLEVCIIGFGFSAIPLVRELARTQTEFQIISAESGSVWDRLSESGRLDFSLVSSFQTSFYSFDLVRDYEKDYYPTAKQFYEMHERWRSVYEEKIIRDFVTKIENFKDYSLISTRSGKTYEAKHVVLATGFDRLMNTFLSNFDNHVSNKTFVFDTMGDSANLLIAKLIPNNNKIILRTNGFTALDQEVQVLGKPFTLDQLESPNFRYVSSELYDRLMMSPVYPRTVNPAVSYNQFPLIRRDFSWVDSKSSPPNGLIAIKYWPIDQYYYHFNDDLENYISKGYLLNDIAMWLHTGKVILVPSDTPINFDKKTITYAGIERSFHQYVKGDAEQPRLPTILINGETPFEYLYRDTFMGVIPQRLNNIYFLGYTRPFTGGLANITEMQSLFIHKLITQPQFHQKIHQNLSKRITAYNQHYYGAAKPRKHDHTVPFGFYTEDIARLIGIHYQPNECRSVRDLLFYYAFPNNAFKYRLKGEYAVDGVDELIQKVNDKHDHYAQVFVQALSIRNMNSDEAAEWDHSARRFSFNDMRHKEGYRAFLDTYLKAYRQVENISVDDTVVDEEWNFMVKEACQVRDKVAPNIEEKTHYSKDEDVNKGIRLILSILDSDISSLPDSNGSRGSGNLKEGDRLCKFEAQSIEFIRRLLQPKNYELLFIRESTVSPGSHRHGETA |

Green, thioredoxin 1; orange, thrombin site; blue, S-Tag; purple, enterokinase site; red, His_6_ tag; underlined, tabaco etch virus cleavage site; reduced-size, (AG)_5_ linker; shaded, AetD and AetF.

**Supplementary Table 2. Mutagenesis oligonucleotides**

| Mutant | Forward primer (5’ - 3’)* |
| --- | --- |
| E75A_F | CATACCCAGGCAGATGCCAAACATTGGGAA |
| D76A_F | ACCCAGGAAGCTGCCAAACATTGGGAATGG |
| H79A_F | GATGCCAAAGCTTGGGAATGGTTTCTGAAT |
| E140A_F | GAAGCAATGGCAACCGCAGGCCTGGTGATT |
| H172A_F | GCCGATAGCGCTGTGGAAGTGGAAACCGGC |
| E176A_F | GTGGAAGTGGCAACCGGCCATGCCGTGGGC |
| H179A_F | GAAACCGGCGCTGCCGTGGGCACAGAAAAT |

*The forward primers are shown and the other complementary primers are omitted here. Underlined, mutated site.

**References**

1. Zhang B*, et al.* Substrate-triggered formation of a peroxo-Fe2(III/III) intermediate during fatty acid decarboxylation by UndA. *Journal of the American Chemical Society* **141**, 14510-14514 (2019).

2. McBride MJ*, et al.* Substrate-triggered μ-peroxodiiron(III) intermediate in the 4-chloro-l-lysine-fragmenting heme-oxygenase-like diiron oxidase (HDO) BesC: Substrate dissociation from, and C4 targeting by, the intermediate. *Biochemistry* **61**, 689-702 (2022).

3. Liu J, Wang Z, Sang X, Zhang X, Wang B. Peroxo-Diiron(III/III) as the reactive intermediate for N-hydroxylation reactions in the multidomain metalloenzyme SznF: Evidence from molecular dynamics and quantum mechanical/molecular mechanical calculations. *ACS Catalysis* **13**, 5808-5818 (2023).
